# Supplementary material for: Fermentative Production of Diacylglycerol by Endophytic Fungi Screened from Taxus chinensis var. mairei
Source: Foods. 2023 Jan 14;12(2):399. doi: 10.3390/foods12020399 (PMC9857645; doi:10.3390/foods12020399)
Supplement: Supplementary file 1 [file foods-12-00399-s001.zip › foods-2071847-supplementary.pdf]

Fermentative Production of Diacylglycerol by Endophytic Fungi Screened from *Taxus chinensis var. mairei*

Wenqiang Xu [a, b], Haoran Bi [a, b], Hong Peng [a, b*], Ling Yang [a, b], Hongwei He [a, b], Guiming Fu [a], Yuhuan Liu [a, b], Yin Wan [a]

[1] *State Key Laboratory of Food Science and Technology, Nanchang University, Nanchang, 330047, P R. China;*

[2] *Engineering Research Center of Biomass Conversion, Ministry of Education, Nanchang University, Nanchang, 330047, P R. China.*

Table S1. Distribution of endophytic fungi in different tissues of *T. chinensis*.

Tissues	Number of endophytic fungi strain	Percentage (%)
Barks	7	18.42
Leaves	9	23.68
Stems	5	13.16
Roots	17	44.74

Table S2. CDW, crude lipid yield, and TLC results of lipid extracts after fermentation for 7 days at 28 °C with shaking speed of 120 rpm.

Tissues	Code	CDW (g/L)	Crude lipid yield (g/L)	TLC results of lipids		
				TAG	1,3-DAG	1,2-DAG
Barks	MLP1N1	9.06	1.40	*****	**	**
	MLP12	4.82	1.69	*****	**	**
	MLP21	7.39	0.79	*****	***	***
	MLP22	2.75	0.88	*****	*	***
	MLP31	4.30	1.70	*****	-	***
	MLP32	8.09	1.27	*****	**	***
	MLP41	5.09	1.94	*****	*****	*****
Leaves	MLY11	10.92	0.88	*****	*	***
	MLY23	9.88	1.45	*****	*****	*****
	MLY32	7.23	0.890	*****	*****	*
	MLY33	5.07	0.28	*****	****	-
	MLY41	2.26	0.12	*****	-	-
	MLY42	6.47	0.49	*****	****	-
	MLY43	7.11	0.60	*****	**	-
	MLY12W	15.29	1.53	*****	***	-
	MLY31W	9.57	1.43	*****	*****	*****
Stems	MLJ11	6.90	0.58	*****	**	*
	MLJ12	12.33	3.34	*****	-	***
	MLJ21	11.62	0.98	*****	**	*-
	MLJ31	8.67	0.45	*****	***	*****
	MLJ33	6.12	0.40	*****	***	*****
Roots	MLG32W	3.08	0.15	*****	*	*****

	MLG32Y	4.14	0.13	*****	*	****
	MLG11	0.87	0.07	*****	*	-
	MLG12	3.85	0.21	*****	*	**
	MLG21	0.93	0.10	*****	*	-
	MLG23	6.86	0.90	*****	****	*****
	MLG31	11.67	0.64	*****	-	****
	MLG32	8.44	0.83	*****	****	**
	MLG42	6.97	0.17	*****	*	*****
	MLGP11	6.56	1.41	*****	*****	*****
	MLGP12	3.65	0.79	*****	-	-
	MLGP13	2.30	1.12	*****	-	***
	MLGP23	7.07	1.44	*****	***	***
	MLGP31	3.62	0.43	*****	***	****
	MLGP33	3.38	0.64	*****	**	***
	MLGP41	0.39	0.12	*****	-	-
	MLGP42	1.79	0.23	*****	*	-

Note: "-" meaning that no TAG band or DAG band was observed; "*" meaning that there was TAG band or DAG band observed, and there was more TAG or DAG was produced with the increase of number of "*".

Table S3. The sequencing and splicing results of the five endophytic fungi

Code	Sequencing and splicing results
MLP41	1 ctgcggaggg atcattaccg agtttacaac tcccaaaccc ctgtgaacat accaattgtt<br>61 gcctcggcgg atcagcccgc tcccggtaaa acgggacggc ccgccagagg acccctaaac<br>121 tctgtttcta tatgtaactt ctgagtaaaa ccataaataa atcaaaactt tcaacaacgg<br>181 atctcttggt tctggcatcg atgaagaacg cagcaaaatg cgataagtaa tgtgaattgc<br>241 agaattcagt gaatcatcga atctttgaac gcacattgcg cccgccagta ttctggcggg<br>301 catgcctgtt cgagcgtcat ttcaaccctc aagccctcgg gtttggtgtt ggggatcggc<br>361 gagcccttgc ggcaagccgg ccccgaaatc tagtggcggt ctcgctgcag cttccattgc<br>421 gtagtagtaa aaccctcgca actggtacgc ggcgcggcca agccgttaaa cccccaactt<br>481 ctgaatgttg acctcggatc aggtaggaat acccgctgaa cttaagcata t
MLG23	1 gagggatcat taccgagttt acaactccca aacccaatgt gaaccatacc aaactgttgc<br>61 ctcggcgggg tcacgccccg ggtgcgtcgc agccccggaa ccaggcgccc gccggaggga<br>121 ccaaccaaac tctttctgta gtcccctcgc ggacgttatt tcttacagct ctgagcaaaa<br>181 attcaaaatg aatcaaaact ttcaacaacg gatctcttgg ttctggcatc gatgaagaac<br>241 gcagcgaaat gcgataagta atgtgaattg cagaattcag tgaatcatcg aatctttgaa<br>301 cgcacattgc gcccgccagt attctggcgg gcatgcctgt ccgagcgtca tttcaaccct<br>361 cgaacccctc cggggggtcg gcgttgggga tcgggaaccc ctaagacggg atcccggccc<br>421 cgaaatacag tggcggtctc gccgcagcct ctcctgcgca gtagtttgca caactcgcac<br>481 cgggagcgcg gcgcgtccac gtccgtaaaa cacccaactt tctgaaatgt tgacctcgga<br>541 tcaggtagga atacccgctg aacttaagca ta
MLY23	1 tgcggaggga tcattactga gtttacgctc tataaccctt tgtgaacata cctataactg<br>61 ttgcttcggc gggtagggtc tccgcgaccc tcccggcctc ccgcctccgg gcgggtcggc

	121 gcccgccgga ggataaccaa actctgattt aacgacgttt cttctgagtg gtacaagcaa<br>181 ataatcaaaa cttttaacaa cggatctctt ggttctggca tcgatgaaga acgcagcgaa<br>241 atgcgataag taatgtgaat tgcagaattc agtgaatcat cgaatctttg aacgcacatt<br>301 gcgcccgcca gcattctggc gggcatgcct gttcgagcgt catttcaacc ctcaagctct<br>361 gcttggtgtt ggggccctac agctgatgta ggccctcaaa ggtagtggcg gaccctcccg<br>421 gagcctcctt tgcgtagtaa ctttacgtct cgcactggga tccggaggga ctcttgccgt<br>481 aaaacccccc aattttccaa aggttgacct cggatcaggt aggaataccc gctga
MLY31W	1 tgcggaggga tcattataga gttttctaaa ctcccaaccc atgtgaactt accattgttg<br>61 cctcggcaga agctacctgg ttaccttacc ttggaacggc ctaccctgta gcgccttacc<br>121 ctggaacggc ctaccctgta acggctgccg gtggactacc aaactcttgt tattatattg<br>181 taatctgagc gtcttatttt aataagtcaa aactttcaac aacggatctc ttggttctgg<br>241 catcgatgaa gaacgcagcg aaatgcgata agtaatgtga attgcagaat tcagtgaatc<br>301 atcgaatctt tgaacgcaca ttgcgcccat tagtattcta gtgggcatgc ctgttcgagc<br>361 gtcatttcaa cccttaagcc tagcttagtg ttgggagcct actgcttttg ctagcggtag<br>421 ctcctgaaat acaacggcgg atctgcgata tcctctgagc gtagtaattt ttatctcgct<br>481 tttgactgga gttgcagcgt ctttagccgc taaacccccc aatttttaat ggttgacctc<br>541 ggatcaggta ggaatacccg ctgaacttaa gcata
MLGP11	1 ccttccgtag gtgaacctgc ggaaggatca ttaccgagtg agggccctct gggtccaacc<br>61 tcccacccgt gtttattcta ccttgttgct tcggcgggcc cgcctcacgg ccgccggggg<br>121 gcacccgccc ccgggcccgc gcccgccgaa gacaccattg aactctgtct gaagattgca<br>181 gtctgagcag attagctaaa tcagttaaaa ctttcaacaa cggatctctt ggttccggca<br>241 tcgatgaaga acgcagcgaa atgcgataag taatgtgaat tgcagaattc agtgaatcat<br>301 cgagtctttg aacgcacatt gcgccccctg gtattccggg gggcatgcct gtccgagcgt<br>361 cattgctgcc ctcaagcacg gcttgtgtgt tgggcttcgc cccccggctc ccggggggcg<br>421 ggcccgaaag gcagcggcgg caccgcgtcc ggtcctcgag cgtatggggc ttcgtcaccc<br>481 gctctgtagg cccggccggc gcccgccggc gaccccaatc aatcttttca ggttgacctc<br>541 ggatcaggta gggatacccg c

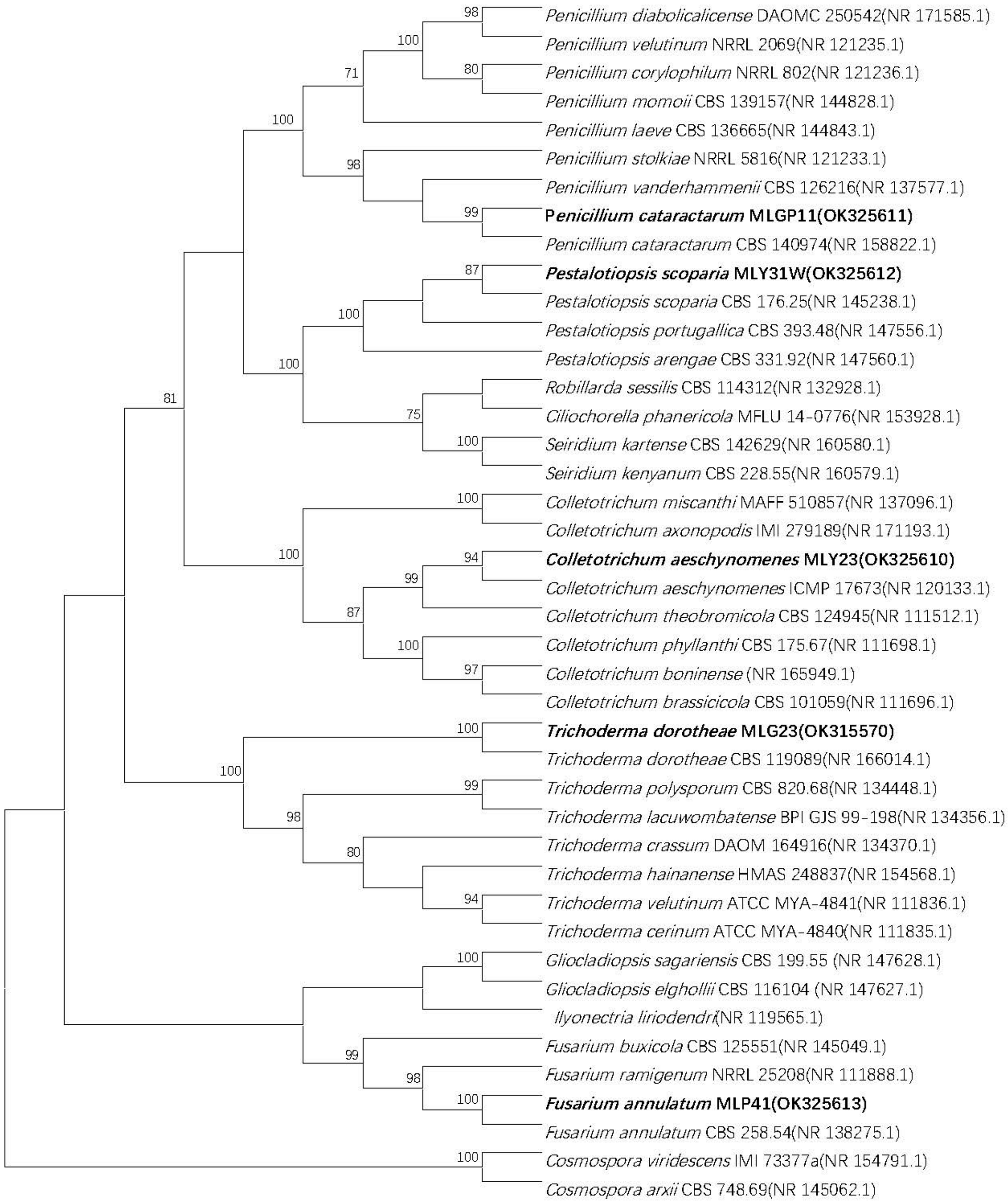

Figure S1. Phylogenetic tree of ITS sequences of endophytic fungi strains coded as MLP41, MLG23, MLY23, MLY31W, and MLGP11 (Gen Bank accession numbers of strains are in brackets).

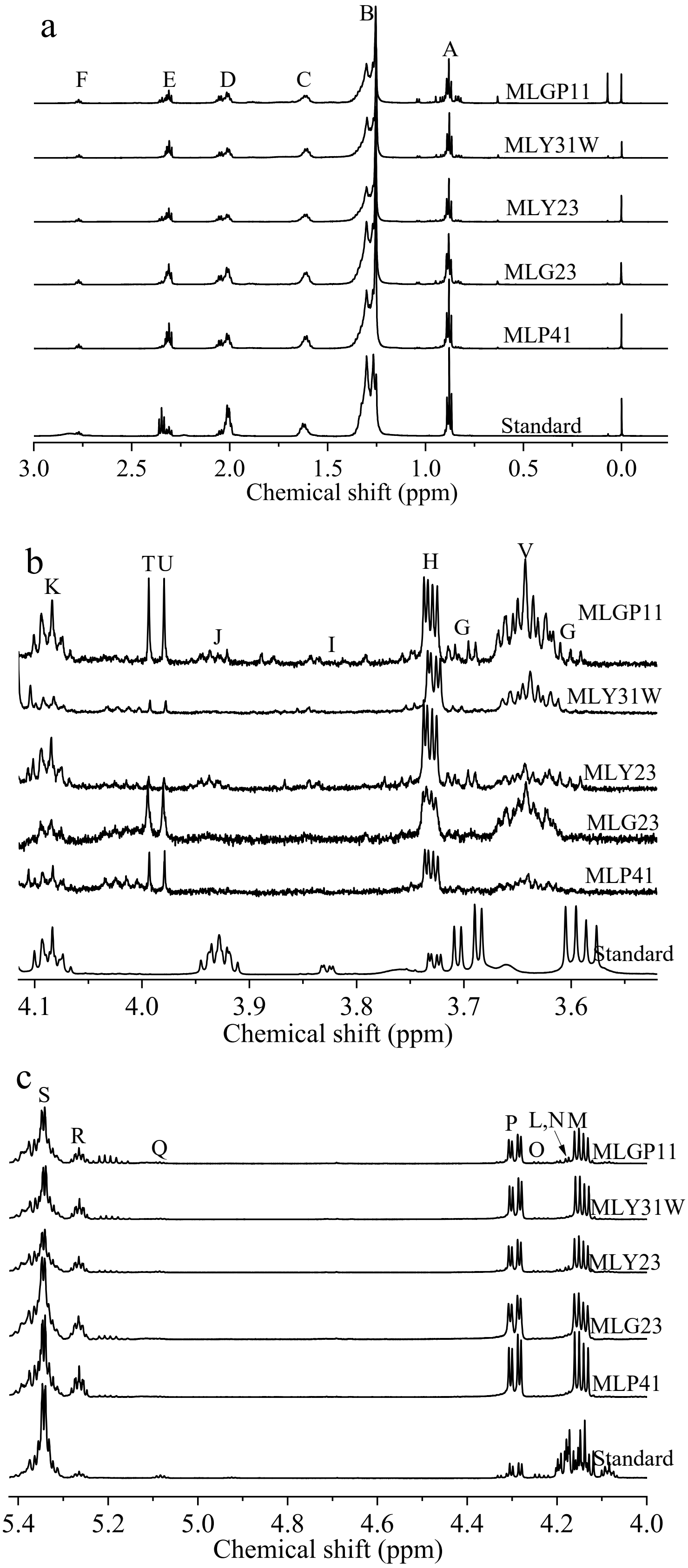

Figure S2. ^{1}H NMR spectra of standards (containing TAG, 1,2-DAG, 1,3-DAG, and MAG) and lipids from MLP41, MLG23, MLY23, MLY31W, and MLGP11. (Solvent $CDCl_3$).
